# Academy of Medicine Notes

**Published:** 1894-08

**Authors:** 


### Academy of Medicine Notes.

THE treasurer's report, read at the annual meeting, showed an excellent condition of the Academy finances.

THE officers of the Academy for 1894–1895 are: President, P. W. VanPeyma, M. D.; vice-presidents, C. C. Wyckoff, M. D., Marcell Hartwig, M. D., M. D. Mann, M. D., S. Y. Howell, M. D.; secretary, A. L. Benedict, M. D.; treasurer, E. A. Smith, M. D.; trustees, Roswell Park, M. D., F. W. Bartlett, M. D., H. R. Hopkins, M. D.; curator of museum, F. S. Metcalfe, M. D.; librarian, F. W. Bartlett, M. D.; council, the above mentioned officers with the secretaries of the different sections—Drs. Sherman, Gilray and Metcalfe—constitute the council of the Academy.

